# The complete chloroplast genome of a moss Korea *Myuroclada maximowiczii* (G.G. Borshch.) Steere & W.B. Schofield

**DOI:** 10.1080/23802359.2020.1823265

**Published:** 2020-10-05

**Authors:** Yeong-Deok Han, Youngeun Choi, Rae-Ha Jang, Young-Jun Yoon

**Affiliations:** a Research Center for Endangered Species, National Institute of Ecology, Yeongyang-gun, Korea; b Department of Ecology Landscape Design, Jeonbuk National University, Jeonju, Korea

**Keywords:** *Myuroclada maximowiczii*, moss, complete chloroplast genome

## Abstract

The complete chloroplast (cp) genome sequence of *Myuroclada maximowiczii* (GenBank accession number MT726030), a species of moss in the Brachytheciaceae family, was determined using Illumina HiSeq paired-end sequencing data. The total size of the cp genome was 124,607 bp. The genome contained a large single-copy (LSC) region of 86,684 bp, a small single-copy (SSC) region of 18,483 bp, and a pair of identical inverted repeat regions (IRs) of 9,720 bp. The genome contained 82 protein-coding genes, 36 transfer RNA (tRNA) genes, and eight ribosomal RNA (rRNA) genes. The cp genome presented here will provide useful information for future phylogenetic and evolutionary studies of *Myuroclada* species.

The genus *Myuroclada* consists of three species worldwide (Crosby et al. 1999; Li et al. 2014), one of which is *Myuroclada maximowiczii* (G.G. Borshch.) Steere & W.B. Schofield. This moss species is distributed in China, Japan, Korea, Russia, Europe, and North America (Hu et al. 2008). The leaves are rounded or ovate, strongly concave, and densely imbricate. The stem and branches are smooth and suggestive of mouse tails (Choe 1980). It forms dense colonies and grows on the base of tree trunks, on rocks, and on wet soil (Choe 1980; Noguchi 1991).

Moss samples were collected from a 3 × 3 cm patch of *M. maximowiczii* from a population growing under natural conditions in Yeongyang-gun County, Gyeongsangbuk-do Province (36°38′50.41″N; 129°9′13.94″E) on 13 January 2020. The specimen was deposited into the JNU Herbarium in Jeonju, Korea, with the accession number YYJ 20200105-1.

Genomic DNA was extracted from fresh moss using a DNeasy^®^ Plant Mini Kit (Qiagen, Hilden, Germany). Illumina HiSeq sequencing (paired-end, 150 bp), *de novo* assembly, and gene annotation were performed according to the methods described by Choi et al. (2020).

The chloroplast (cp) genome of *M. maximowiczii* was found to have a total length of 124,607 bp, containing 126 genes in total, including 82 protein-coding genes (PCGs), 36 transfer RNA (tRNA) genes, and 8 ribosomal RNA (rRNA) genes. The gene arrangement of the *M. maximowiczii* cp genome was found to be similar to that of the traditional bryophyte cp genome. The cp genome of *M. maximowiczii* was found to have 30.1% G + C content, and the base composition was as follows: A = 34.8%, C = 15.1%, G = 15.0%, and T = 35.0%. The cp genome consisted of a large single-copy region (LSC) of 86,684 bp and a small single-copy (SSC) region of 18,483 bp separated by a pair of inverted repeat (IR) regions of 9720 bp.

We inferred the phylogenetic relationship of *M. maximowiczii* based on ten bryophyte cp genomes including *M. maximowiczii* and three outgroup species (two Marchantiophyta and one Anthocerotophyta). Concatenated sequences of 16 PCGs were used for constructing a bootstrapped maximum likelihood tree using MEGA X method based on the JTT matrix-based model with 1000 bootstrap replicates (Kumar et al. 2018) (Figure 1).

**Figure 1.**
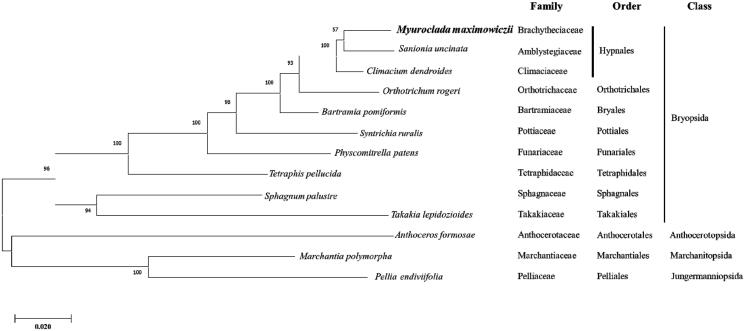
Phylogenetic position of *M. maximowiczii* determined by Maximum likelihood methods based on combined analysis with amino acids sequences of 16 chloroplast genes common in all taxa. Bootstrap values over 50% from 1000 replicates are exhibited for corresponding branches. Sequences from 1 Marchantiopsida, 1 Jungermanniopsida and 1 Anthocerotophyta were used as outgroup. GenBank accession numbers of chloroplast genomes used are *Anthoceros formosae* (NC_004543), *Bartramia pomiformis* (MT024676), *Climactium dendroides* (MT006132), *Marchantia polymorpha* (NC_037507), *Myuroclada maximowiczii* (MT726030), *Orthotrichum rogeri* (NC_026212), *Pellia endiviifolia* (NC_019628), *Physcomitrella patens* (NC_037465), *Sanionia uncinata* (NC_025668), *Sphagnum palustre* (NC_03019), *Syntrichia filaris* (MK852705), *Takakia lepidozioides* (NC_028738) and *Tetraphis pellucida* (NC_024291).

The cp genome of *M. maximowiczii* can provide a reference for further studies on the phylogeny and evolution of the genus *Myuroclada*. Additionally, the cp genome sequence can be used for species identification or confirmation, and information about the *M. maximowiczii* cp genome will be useful for evolutionary studies of organelle genomes of the Bryophyta, including Korean moss species.

## Data Availability

The data that support the findings of this study are openly available in GenBank of NCBI at https://www.ncbi.nlm.nih.gov, reference number MT726030.
